# Spontaneous Regression of Metastatic Colorectal Cancer Following Discontinuation of an Interleukin-6 Receptor Inhibitor: A Case Report

**DOI:** 10.70352/scrj.cr.25-0710

**Published:** 2026-03-11

**Authors:** Eiki Miyake, Fumitaka Taniguchi, Mikoto Nosaka, Kengo Mouri, Maho Sato, Hiroki Kajioka, Toshihiro Ogawa, Megumi Watanabe, Koh Katsuda, Akiko Iseki, Yumiko Sato, Kohji Tanakaya, Hideki Aoki

**Affiliations:** 1 Department of Surgery, National Hospital Organization Iwakuni Clinical Center, Iwakuni, Yamaguchi, Japan; 2 Department of Pathology, National Hospital Organization Iwakuni Clinical Center, Iwakuni, Yamaguchi, Japan

**Keywords:** spontaneous cancer regression, colorectal cancer, immunosuppressive therapy discontinuation

## Abstract

**INTRODUCTION:**

Spontaneous cancer regression is exceptionally rare, particularly in colorectal cancer. Tumor regression has been reported in some patients with autoimmune diseases or organ transplants, after cessation of immunosuppressive therapy; however, its underlying mechanisms remain unclear. We report a rare case of Stage IVA transverse colon cancer with synchronous liver metastasis that showed complete pathological regression in both sites after discontinuation of tocilizumab, an interleukin-6 receptor inhibitor.

**CASE PRESENTATION:**

A 79-year-old woman with a history of rheumatoid arthritis and interstitial pneumonia was treated with iguratimod, methotrexate, prednisolone, and tocilizumab. PET-CT images revealed an accumulation of fluorodeoxyglucose in the transverse colon and liver. Colonoscopy and biopsy findings confirmed a poorly differentiated adenocarcinoma in the transverse colon; liver biopsy findings confirmed metastatic disease. In preparation for surgery, and because the rheumatoid arthritis activity was stable, tocilizumab was discontinued. Three months after tocilizumab discontinuation, laparoscopic partial colectomy with lymph node dissection was performed. Histopathologic examination of the resected colon showed no viable tumor cells. Subsequently, laparoscopic partial hepatectomy of segments 2 and 3 revealed no viable tumor cells as well. The patient remained recurrence-free at the 2-year follow-up.

**CONCLUSIONS:**

The findings of this case suggest that tocilizumab discontinuation may have reactivated antitumor immune responses, resulting in spontaneous regression of both the primary colorectal cancer and liver metastasis. Notably, prior to regression, both masses were histologically confirmed as malignant lesions. This observation provides valuable clinical insight into the relationship between interleukin-6 receptor blockade, immune modulation, and tumor dynamics.

## Abbreviations

CD8^+^ T cell
: cluster of differentiation 8-positive T cell
IL-6
: interleukin-6
MMR
: mismatch repair
MSI
: microsatellite instability
UICC
: Union for International Cancer Control

## INTRODUCTION

Spontaneous cancer regression, which is extremely rare, is defined as the partial or complete disappearance of a malignant tumor without a specific anticancer therapy being administered. Occasionally, in patients with autoimmune diseases or organ transplants, tumors have been reported to regress after cessation of treatment with immunosuppressive drugs. In 1 case, a lung transplant recipient experienced complete regression of colorectal cancer with liver metastases after reduction of immunosuppressive therapy.^1)^ Cases of hepatocellular carcinoma regression after discontinuation of immunosuppressive agent therapy have also been reported.^2)^ Tocilizumab, a humanized monoclonal antibody that acts against the IL-6 receptor, is widely used to treat rheumatoid arthritis; however, its effects on tumor immunity remain unclear. Herein, we report a rare case of Stage IVA transverse colon cancer with synchronous liver metastasis that showed complete pathological regression at both sites after discontinuation of tocilizumab therapy.

## CASE PRESENTATION

**Fig. 1:**
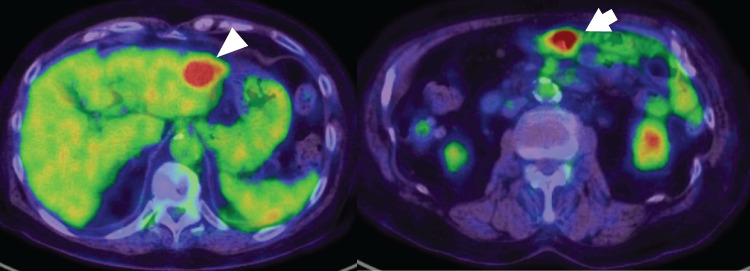
PET-CT image obtained at the initial visit. Fluorodeoxyglucose uptake is observed in the lateral segment of the liver (arrowhead) and transverse colon (arrow).

**Fig. 2:**
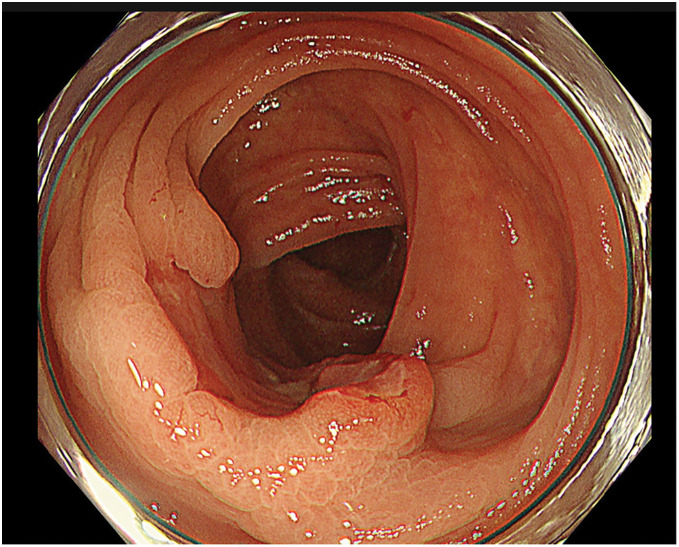
Colonoscopy image 1 month after the initial visit. An ulcerated lesion with clear margins is shown in the transverse colon.

**Fig. 3:**
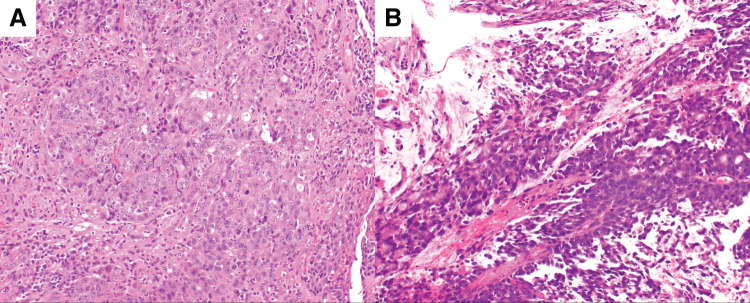
Histological findings of the colon and liver biopsy specimens. (**A**) Histological examination of a colonoscopic biopsy specimen from the transverse colon reveals a poorly differentiated adenocarcinoma (hematoxylin and eosin staining, ×100). (**B**) Histological examination of a liver biopsy specimen from the lateral segment confirms metastasis from colorectal cancer (hematoxylin and eosin staining, ×100).

**Fig. 4:**
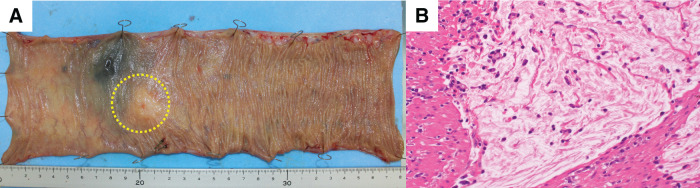
Histological findings of the resected transverse colon specimen. (**A**) Resected transverse colon specimen, with the lesion area visible (dashed-line circle). (**B**) Histological examination revealed fibrosis and lymphocyte infiltration extending from the muscularis propria to the subserosa, with mucin pools but no viable tumor cells (hematoxylin and eosin staining, ×200).

**Fig. 5:**
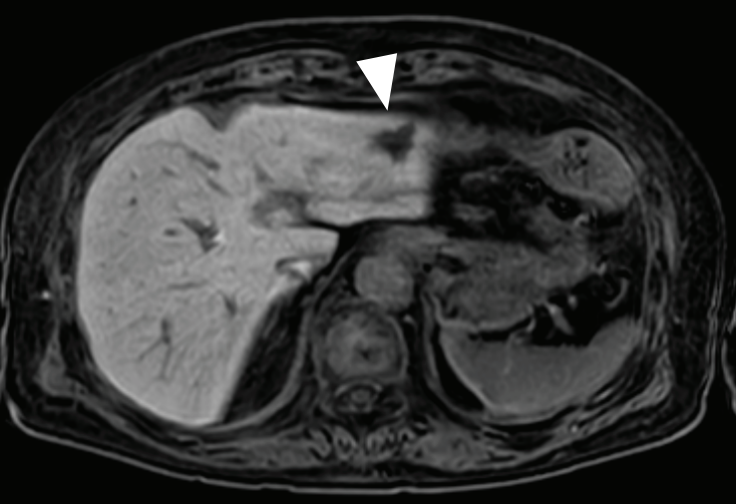
Gadoxetic acid–enhanced MRI performed before hepatectomy. The image demonstrates the identified liver lesion in the lateral segment, showing reduced gadoxetic acid uptake (arrowhead), without evidence of additional hepatic metastases.

**Fig. 6:**
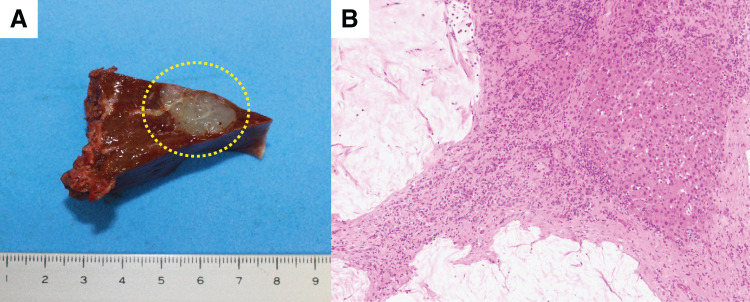
Histological findings of the resected liver specimen. (**A**) Resected liver specimen (segments 2 and 3), with the lesion area visible (dashed-line circle). (**B**) Histological examination revealed mucin pools within the scar tissue, with no viable tumor cells (hematoxylin and eosin staining, ×4).

**Fig. 7:**
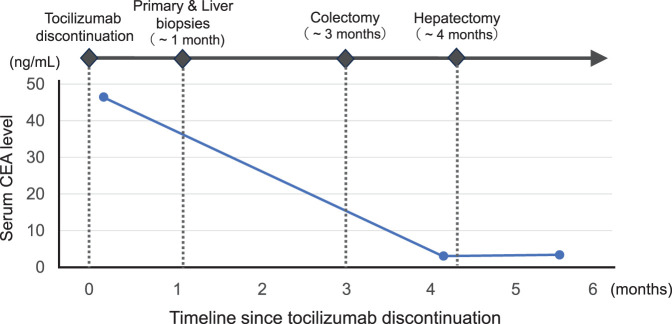
Timeline of clinical events following tocilizumab discontinuation. Tocilizumab therapy was discontinued after the detection of liver metastasis. One month later, colon and liver biopsies confirmed adenocarcinoma and metastasis, respectively. Laparoscopic colectomy was performed 3 months after tocilizumab discontinuation, followed by hepatectomy 1 month later. Both lesions showed complete pathological regression. The line graph shows serial changes in serum CEA levels during this period. CEA, carcinoembryonic antigen

A 79-year-old woman was referred to our facility after the incidental detection of a liver mass on non-contrast CT during a follow-up for interstitial pneumonia. The patient, who was asymptomatic, was receiving treatment for rheumatoid arthritis and interstitial pneumonia. At the time, rheumatoid arthritis symptoms were well-controlled with iguratimod (25 mg), methotrexate (2 mg), prednisolone (1 mg), and tocilizumab (480 mg). Tocilizumab was discontinued after the detection of a liver metastasis at the previous hospital, in anticipation of potential surgical treatment and because rheumatoid arthritis disease activity was stable. The remaining immunosuppressive agents, including iguratimod, methotrexate, and prednisolone, were continued during this period. PET-CT images revealed fluorodeoxyglucose uptake in the lateral segment of the liver and transverse colon (**Fig. 1**). Contrast-enhanced CT was not performed because of impaired renal function. An ulcerated lesion with clear margins was revealed in the transverse colon via colonoscopy (**Fig. 2**), and a poorly differentiated adenocarcinoma was confirmed via biopsy (**Fig. 3A**). A core needle biopsy of the liver lesion was performed, and the findings confirmed liver metastasis from the colon cancer (**Fig. 3B**). These biopsies were performed 1 month after tocilizumab discontinuation. Serum tumor marker biochemistry showed that the levels of carbohydrate antigen 19-9 were within the normal range, whereas those of carcinoembryonic antigen were elevated at 46.5 ng/mL. Based on these findings, the patient was diagnosed with transverse colon cancer with liver metastasis (cT2N1aM1a, cStage IVA, UICC TNM classification, 8th edition). Approximately a timeframe of 1 week was required from the needle biopsy to establish a pathological diagnosis. Although the patient had Stage IVA colorectal cancer with liver metastasis, the liver lesion was solitary and the patient did not exhibit any symptoms related to the primary tumor, such as difficulty in endoscopic passage, anemia, or abdominal pain. Therefore, the clinical condition did not warrant emergency surgery, so an elective surgical approach was deemed appropriate. Owing to limitations in surgical scheduling, the operation was performed approximately 6 weeks after referral to the Surgical Department. Consequently, a period of approximately 2 months elapsed between establishing the diagnosis and surgery. The patient underwent laparoscopic partial colectomy (transverse colon) with D3 lymph node dissection 3 months after tocilizumab discontinuation. The resected specimen showed fibrotic scar tissue at the lesion site (**Fig. 4A**). Histological examination of the primary lesion revealed fibrosis and lymphocytic infiltration, with mucin pools extending from the muscularis propria to the subserosa; however, no viable tumor cells were observed (**Fig. 4B**). As with the primary site, the dissected lymph nodes also contained mucin pools, but no viable tumor cells were identified. Before hepatectomy, gadoxetic acid–enhanced MRI was performed for comprehensive evaluation of the liver metastasis. It demonstrated the known lesion in the lateral segment with reduced gadoxetic acid uptake and no evidence of additional hepatic metastases (**Fig. 5**). One month after colectomy, the patient underwent a laparoscopic partial hepatectomy of segments 2 and 3 of the liver. Histological examination of the resected liver tissue showed mucin pools only within the scar tissue, similar to those in the primary lesion, with no residual tumor cells (**Fig. 6A** and **6B**). Both the primary and liver lesions were sectioned in their entirety for comprehensive histopathological evaluation, in which no viable tumor cells were identified. These findings differed from typical mucinous adenocarcinoma, in which tumor cells are usually observed within mucin lakes. In addition, the specimens were evaluated by multiple pathologists who confirmed the absence of residual tumor. Postoperative adjuvant chemotherapy was not administered. Although the necessity and potential risks of adjuvant treatment were explained in accordance with the current clinical guidelines, the patient preferred not to receive chemotherapy. The chronological relationship between tocilizumab discontinuation, diagnostic procedures, and surgeries is illustrated in **Fig. 7**. No recurrence was observed at the 2-year follow-up, via monitoring with non-contrast-enhanced CT every 6 months.

## DISCUSSION

Spontaneous colorectal cancer regression is exceptionally rare. A comprehensive review of cases reported between 1900 and 2005 identified only 21 patients.^3)^ This extreme rarity may partly be explained by the fact that most colorectal cancers are surgically resected shortly after diagnosis, leaving little opportunity for spontaneous regression to occur or be observed. Furthermore, among 14 cases in which potential contributing factors were described (excluding 7 cases with insufficient data), infection was reported as a possible trigger in 6 cases (42.9%), suggesting that immune responses may play a role in the underlying mechanism.^3)^ However, as many of these reports date back to the early 20th century, the diagnostic accuracy of spontaneous regression in those cases remains uncertain. Unlike some reported cases of cancer regression in which the diagnosis was based on radiological findings alone, the present case is distinct in that both the primary and metastatic lesions were histologically confirmed before treatment and showed complete pathological regression after resection. This dual histological verification provides compelling evidence of true spontaneous regression. To distinguish true regression from other pathological entities, the histological features were compared with those of mucinous adenocarcinoma. Mucinous colorectal adenocarcinoma typically shows abundant extracellular mucin associated with malignant epithelial components, which are generally identifiable even in small amounts.^4)^ In contrast, the present case showed extensive mucin pools completely devoid of tumor cells in both the primary and metastatic lesions. Since the entire lesions were thoroughly examined and no viable tumor cells were detected, these findings are unlikely to represent under-sampling or mucinous differentiation. Rather, the combination of acellular mucin, fibrosis, and lymphocytic infiltration supports complete pathological regression following immune-mediated tumor destruction.

Complete pathological regression of both the primary colorectal tumor and synchronous liver metastasis after tocilizumab discontinuation was observed in the present case, while other immunosuppressive therapy was maintained. Such a clinical course is very rare in colorectal cancer and suggests a possible link between withdrawal of IL-6 blockade and restoration of antitumor immunity. Tocilizumab is a humanized monoclonal antibody that binds to both soluble and membrane-bound IL-6 receptors, preventing IL-6 from activating the downstream JAK/STAT3 pathway; through this mechanism, tocilizumab reduces inflammation.^5)^ IL-6 contributes to the activation and differentiation of immune cells, including rapid effector programming of CD8^+^ T cells.^6)^ IL-6 signaling has also been shown to be essential for the antitumor activity of transforming growth factor-beta–specific T cells in a prostate cancer model, suggesting that IL-6 blockade could limit certain tumor-specific immune responses.^7)^

The treatment timeline in the present case supports this hypothesis. The patient had been undergoing treatment with iguratimod, methotrexate, prednisolone, and tocilizumab for rheumatoid arthritis. Iguratimod, methotrexate, and prednisolone were continued during the period when the tumor regressed, while only tocilizumab was discontinued 3 months before surgery for the primary lesion. This sequence suggests that the change in immune activity was related to tocilizumab cessation. Although a direct causal relationship cannot be established, the temporal association is notable.

We also considered other possible causes of tumor regression. Infection,^3,8,9)^ inflammation,^10)^ ischemia,^10)^ surgical trauma,^11)^ and medications^11)^ have been proposed as triggers for spontaneous regression. In the present case, biopsy-induced immune activation has also been proposed as a potential trigger for spontaneous regression^8)^ and cannot be ruled out. In addition, spontaneous regression has been reported more frequently in patients with dMMR/MSI-H colorectal cancers.^12,13)^ Herein, molecular profiling, including MSI/MMR status and RAS/BRAF mutations, was not performed, precluding consideration of this possibility. Preoperative biopsy specimens were insufficient for genomic testing, as upfront curative resection was prioritized without planned neoadjuvant therapy. Postoperatively, molecular evaluation was not feasible because no viable tumor cells were identified in the resected specimens due to complete tumor regression. Immunological assessments such as PD-L1 expression, tumor-infiltrating lymphocytes, and tumor mutational burden were not performed.

The present case has important clinical implications for managing autoimmune diseases in patients with cancer. Several large-scale studies have evaluated the safety of tocilizumab, including increased malignancy risk. For example, the STREAM trial, involving patients with early rheumatoid arthritis, found that the incidence of malignancy in the tocilizumab group was comparable to that in the general population and in patients with rheumatoid arthritis.^14)^ Similarly, the LITHE trial demonstrated that tocilizumab combined with methotrexate significantly inhibited joint damage progression, with no significant increase observed in malignancy incidence.^15)^ However, these findings do not guarantee absolute safety; malignancies may still develop during tocilizumab therapy, as in the present case, and recognizing that tumor regression might occur after its discontinuation is important. The practice guidelines for rheumatoid arthritis in Japan^16)^ state that the long-term safety of biologic disease-modifying antirheumatic drugs, including tocilizumab, is uncertain in patients with a history of malignancy, and suggest care when making treatment decisions.

A few cases have been reported in which tumor regression occurred following the withdrawal or dose reduction of immunosuppressive agents. However, to our knowledge, this is the first reported case worldwide in which complete disappearance of colorectal cancer was observed after discontinuing an anti-IL-6 antibody. We report this case because it provides important insight into the potential antitumor effects of immune activation, as well as the possible role of immunosuppression in tumor development and progression. The findings of this case confirm the interplay between immune modulation and tumor behavior, highlighting both the potential therapeutic implications of immune activation and the oncogenic risks associated with immunosuppression.

## CONCLUSIONS

In the present patient, tocilizumab discontinuation was temporally associated with complete pathological regression of both the primary colorectal cancer and synchronous liver metastasis. Although causality could not be established, the case highlights the potential effect that IL-6 blockade has on tumor-immune dynamics. Future studies are needed to elucidate the means by which IL-6–related immunosuppression and immune reactivation influence tumor-immune dynamics.
